# The Use of Medicinal Leeching in Breast Surgery: A Systematic Review

**DOI:** 10.3390/jcm13051243

**Published:** 2024-02-22

**Authors:** Rohan Rajaram, Jevan Cevik, Nayan Bhindi, Ishith Seth, Warren M. Rozen

**Affiliations:** 1 Department of Plastic and Reconstructive Surgery, Peninsula Health, Frankston 3199, Australia; 2 Peninsula Clinical School, Central Clinical School, Faculty of Medicine, Monash University, Frankston 3004, Australia

**Keywords:** breast reconstruction, leeches, hirudotherapy, flap salvage

## Abstract

**Background:** The medicinal leech has been used in plastic surgery to resolve venous congestion that can threaten the viability of tissue transfer. Within the context of breast surgery, venous congestion is a pertinent consideration for reconstructive and non-reconstructive breast surgery such as mammoplasty and mastopexy. However, leeching is closely associated with complications such as infection, pain, and anaemia. This is the first systematic review that examines the methodology, efficacy, and post therapeutic outcome data across all existing studies on medicinal leeching in breast surgery. **Methods:** A systematic search of PubMed and Embase databases from their inception to November 2023 was conducted. Inclusion criteria included studies reporting on the use of leeches to resolve venous congestion in any breast surgery. The JBI Critical Appraisal Checklist for Case Series tool was used for bias analysis. Descriptive statistics were undertaken in Microsoft Excel. **Results:** A total of 18 studies with a combined sample size of 28 were examined, including 4 case series and 14 case reports. Patients mostly underwent reconstructive breast surgery (75%). The median number of leeches used was two, with a median number of three leeching sessions per day and 3 days of leeching. Medicinal leeching successfully prevented the loss of 75% of all tissue transfers. The complication rate was high at 81.14% and mainly included infection and anaemia. **Conclusions:** Medicinal leeching is an effective method to relieve venous congestion in breast surgery but must be judiciously used within the clinical context of the patient to maximise efficacy and mitigate harm from complications.

## 1. Introduction

The popularity of leeches in medicine has varied as our conceptualisation of disease has evolved with time. During the time of Galen (129–200 AD), an imbalance in the humour of blood necessitated exsanguination to cure the ailment [1]. While in modern times such rudimentary utilisation of leeches has all but been discredited, there still exist some specific medical and surgical uses for the medicinal leech [2]. Most prominently, *hirudotherapy,* the use of the medicinal leech (*Hirudo Medicinalis*), is harnessed by reconstructive surgeons to relieve venous congestion in free and pedicled flaps, as well as in replantation procedures [3]. Venous insufficiency, through many aetiologies, is one of the most common causes of failure of tissue transfer. The uninhibited accumulation of venous blood ultimately leads to arterial insufficiency, tissue necrosis, and flap loss. Furthermore, venous insufficiency is especially hostile to the survival of a flap due to the microcirculatory changes it causes such as platelet trapping and microthrombi formation [4].

Many procedures in breast surgery suffer from venous insufficiency. In addition to the regular use of reconstructive techniques such as the pedicled flaps (e.g., pedicled trans rectus abdominis myocutaneous flap) and free flaps (e.g., deep inferior epigastric artery perforator flap), non-reconstructive breast surgery is also threatened by compromised venous supply. For example, in mammoplasty and mastopexy, moving the vascular pedicle supplying the nipple areolar complex (NAC) to its new position may compromise venous patency resulting in a congested NAC [5]. Therefore, in breast surgery, venous insufficiency can be caused either by thrombosis of the anastomosed pedicle, a compromise to the pedicle supplying the NAC, or by a more distal venous insufficiency at the level of the cutaneous venosome of the flap. To combat this, the medicinal leech provides two active mechanisms by which venous congestion is relieved: 1. active extraction of blood from the tissue and 2. the salivary release of anticoagulative and vasoactive substances such as hirudin and histamine which promote the drainage of blood for hours after the leech has detached [6]. This can temporise the tissue for long enough to form new venous networks to bolster drainage.

However, medicinal leeching is not without its drawbacks. In the general plastic surgery realm, the use of leeches brings with it complications of pain, infection, and patient reluctance. Furthermore, it stands to reason that proximal venous failure as in thrombosis or proximal pedicle torsion/kinking may not respond as favourably to a distally based technique such as leeching [7]. These considerations may yet again bring into question the role of leeches in breast surgery and whether they provide a net benefit to the patient and surgeon alike. Despite their widespread use in plastic and reconstructive surgery, literature on hirudotherapy in breast surgery is notably scarce. Unlike other areas of plastic surgery with comprehensive reviews like that of Whittaker et al. [3], research in breast surgery primarily consists of a few case reports and series, lacking any controlled studies. This systematic review seeks to gather and analyse all existing data on medicinal leeching in breast surgery, identifying typical uses, application methods, effectiveness, and potential complications, thereby guiding clinicians in their practice.

## 2. Methods

**Figure 1:**
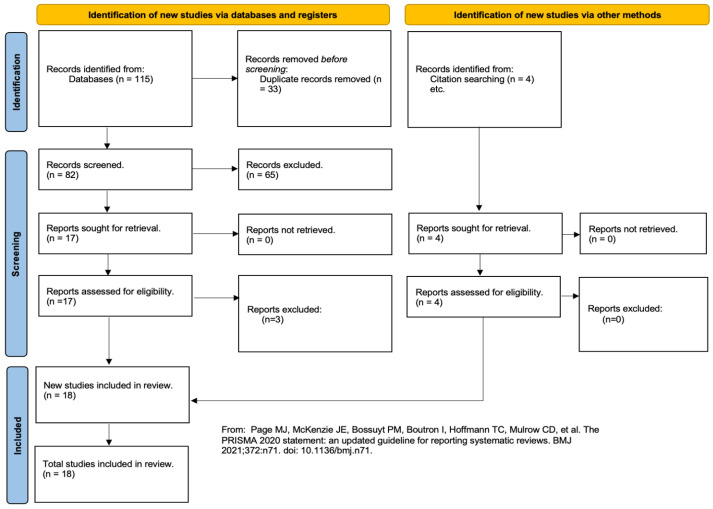
PRISMA flow diagram of study inclusion [9].

This systematic review adhered to the Preferred Reporting in Systematic Review and Meta-Analysis (PRISMA) guidelines (Figure 1) and was listed prospectively on the International Prospective Register of Systematic Reviews (PROSPERO) [8]. Pubmed and Embase Library were thoroughly searched from inception until November 2023. The search strategy utilised a combination of relevant keywords and MeSH terms. The following search terms were used: (Medicinal Leeching OR Leech* OR Hirudo Med* OR Medical Leech Therapy) AND (Breast OR Breast Reconstruction OR Nipple OR Nipple Areolar Complex OR Oncoplas* OR Mammoplast* OR Mastopex*). During the review process, any relevant studies discovered through references were also manually added. All papers were independently reviewed by two authors (RR and JC). Discrepancies that arose during either the title and abstract screening or the full text review were discussed and resolved involving a third reviewer when necessary.

### 2.1. Study Inclusion

Inclusion criteria were the following: any study that described the use of leeching on one or more patients undergoing breast surgery. The studies were required to explicitly describe the indication for, technique of, and endpoints of leeching and describe any complications and medical or surgical adjuvants used. Given the significant lack of higher sample size studies on this relatively niche area, case reports and case series were included in this review. Studies investigating the use of leeches in other surgical contexts, conference abstracts, review articles, and letters to the editor were not included. This review was limited to English language studies on human subjects.

### 2.2. Data Extraction

Title and abstract screening were independently undertaken by two reviewers (RR and JC) and discrepancies were resolved through discussion and the involvement of a third reviewer. Data were extracted into data extraction tables and multiple data points were analysed to provide a comprehensive review of the literature. The following information about the studies was extracted: author, year of publication, country of study origin, and type of study. Additionally, the following independent variables were extracted: sample size, patient age, procedure performed, indication for hirudotherapy, prior attempts at resolving venous congestion, number of leeches used per session, number of leeching sessions per day, and total length of hirudotherapy. The primary outcome investigated was the success or failure of hirudotherapy in resolving the venous congestion and the following additional endpoints were gathered: complication type, causative micro-organism in the instance of infection, pharmacological adjuvants in the resolution of venous congestion and hirudotherapy-related complications (excluding prophylactic antibiotics), and units of packed red blood cells (pRBCs) in cases of anaemia.

### 2.3. Quality Assessment

The emerging nature of this research resulted in much of the literature being comprised of case studies and series. Thus, it was decided that the Joanna Briggs Institute (JBI) Critical Appraisal Checklist for Case Series [8,10] could be used to appropriately qualify studies based on their risk of bias. The checklist consists of 10 questions which cover the investigative rigour and potential for bias within the inclusion criteria, subject identification, patient reporting and statistical analysis used in single arm studies. Each question could be answered as Yes, No, Unclear, or Not Applicable. This quality assessment was undertaken by RR and JC and disagreements were resolved through discussion and the inclusion of a third reviewer.

### 2.4. Quantitative Analysis

The case reports and series that predominated our included studies described their outcomes without measures of spread (and with no reasonable way of calculating the same). This combined with the low individual sample size of many of the papers resulted in meta-analysis methods being an inappropriate statistical approach for this topic. Instead, descriptive statistical methods were utilised. Numerical variables were expressed as means with a range and categorical variables were expressed as both absolute and relative frequencies. Odds ratios and Fisher tests describing the associations between two variables were conducted, with statistical significance being set as a *p* value of <0.05. These calculations were undertaken in Microsoft Excel version 16.82.

## 3. Results

### 3.1. Literature Search

Out of 115 identified results, 33 duplicates were removed and 82 underwent title and abstract screening. Of these, 65 were removed and 17 underwent full text screening. During full text screening, four papers were discovered through reference searching and were included manually. Ultimately, 18 papers were included in this systematic review (Figure 1) [9].

### 3.2. Study Characteristics

The included studies were published between 1984 and 2022. Of the 18 included studies, 14 were case reports and 4 were case series. Sample sizes ranged from 1 to 6 with a total sample size of 28. Not all studies reported on all analysed parameters. All reported the operations undertaken, indications for hirudotherapy, and its success rate in resolving venous congestion; 17 reported the total period of hirudotherapy as well as any encountered complications; 15 reported the ages of patients and pharmacological adjuvants; 14 reported prior attempts of resolving venous congestion; 12 reported the number of leeches; 7 reported the frequency of leeching; and only 2 reported the number of pRBCs used in the resolution of anaemia (Table 1 and Table 2).

### 3.3. Quality Assessment

In the context of single-arm case series and single-patient case reports, most studies demonstrated a low risk of bias as evaluated by the JBI checklist (Table 3). The largest source of bias was in the completeness of participant inclusion (Q5). Most case series had stringent inclusion criteria due to the niche role of leeching in breast reconstruction and, as a result, limited the patients that were selected for inclusion. Furthermore, while the completeness of patient inclusion is not discernible in case reports, it still introduces an uncertain level of bias to each paper. Finally, another common source of bias was in the sometimes incomplete reporting of the clinical information of patients (Q7). Some papers such as Ardehali et al. and Moffat et al. only included clinical patient details that were related to their breast surgery, thereby partially narrowing the scope through which their reported data could be interpreted. However, these minor sources of bias were accepted in an attempt to capture a large sample size.

### 3.4. Patient Characteristics

All 28 patients were biological females. The mean age of patients was 43.4 years (range 23–61). The most common breast surgery performed across all studies was free flaps for breast reconstruction (62.5%) followed by pedicled flaps (20%), with non-reconstructive procedures falling in the minority (reduction mammoplasty = 15% and mastopexy 2.5%).

### 3.5. Indications for Hirudotherapy and Prior Attempts at Salvage

All papers, with a combined sample size of 28, reported indications for medical leech therapy. The most common indication for leeching was the cutaneous portion of a flap (either free or pedicled) experiencing venous congestion (57.1%), followed by the nipple areolar complex (NAC) becoming congested (25%), and finally due to a venous insufficiency caused by venous thrombus in free flap anastomosis (17.9%).

A subset of these papers—14 papers with a sample size of 24—also described any steps taken before leeching to improve venous congestion. Of these, 15 patients (62.5%) underwent leeching with no prior intervention, 7 (29.1%) were taken back to the theatre to re-inset the flap or re-anastomose the pedicle, and 2 (8.3%) were given a pharmacological agent such as GTN, Aspirin, or Pentoxifylline.

### 3.6. Leech Application Protocol

**Figure 2:**
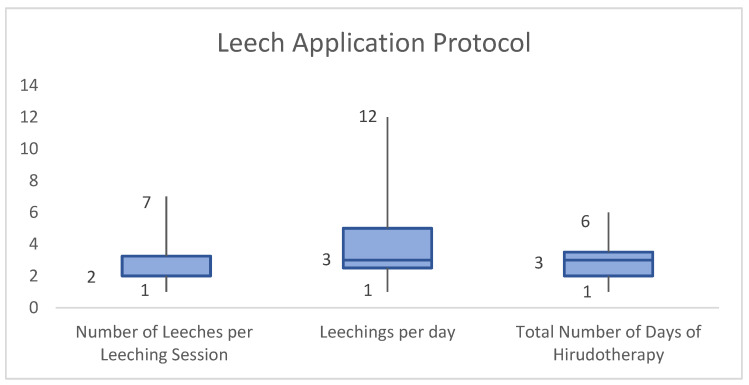
Box plots of number of leeches per leeching, leeching frequency, and total number of days of hirudotherapy.

There were 17 papers with a combined sample size of 27 that reported on the total length that hirudotherapy was undertaken. The median number of days that leeches were applied with varying regularity was 3 (range 1–6). Of these papers, a subset of 12 papers and a sample size of 16 patients also mentioned the number of leeches given per session, which had a median of 2 leeches (range 1–7). A further subset of these studies, seven with sample size of 11, also described the number of times a day leeches were applied. This had a median of 3 times a day (range 1–12) (Figure 2).

### 3.7. Pharmacological Adjuvants

The 17 papers with a sample size of 27 described any pharmacological means used in conjunction with hirudotherapy. A total of 15 out of 27 patients (55.67%) received no additional pharmacotherapy, 9 patients (33.3%) received targeted antibiotic therapy (not prophylactic) for infection, and 1 patient (3.7%) each received a vasodilator, an anticoagulant, and a steroid.

### 3.8. Success Rates

The primary outcome of success was reported in all papers with a sample size of 28. The success of medical leeching in salvage was defined as any outcome other than 100% skin/flap loss, with partial zones of necrosis tolerated and treated as a complication. When success was stratified by indication, venous thrombosis was least responsive to leeching with a 60% success rate, followed by cutaneous venous congestion in the flap which was resolved 68% of the time. All NAC congestions were resolved by leeching. Non reconstructive breast surgery (mastopexy + mammoplasty) cases saw 100% success in the resolution of venous congestion with many studies reporting no skin loss following hirudotherapy. Pedicled flap cases were the next most encouraging with only the Bourdais et al. and one of the De Chalain cases suffering from complete flap loss, a success rate of 75%. Finally, free flap cases saw the greatest level of complete flap loss. Three out of the four Pannucci et al. free flaps, one of the four De Chalain et al. reports, and the Barraud free flap were completely lost. This resulted in a total free flap salvage rate of 61.5%. The overall success rate across all papers was calculated to be 75%.

When comparing salvage free flaps to pedicled flaps, a non-significant odds ratio of success of 1.87 was calculated in favour of pedicled flaps. Furthermore, when venous congestion to venous thrombosis was compared, a non-significant odds ratio of 1.46 was calculated in favour of venous congestion.

### 3.9. Complications

The 17 papers with a sample size of 27 showed that 22 patients experienced 32 complications (Table 1). Given that leech therapy was used to treat flap failure, total and partial flap failure were not considered as direct complications resulting from leeching itself. Therefore, complications resulting from leech therapy amounted to 16 patients of the 27 (59.3%). The most common complication was infection occurring in nine instances, and the next was anaemia requiring blood transfusion, occurring eight times. Finally, a single case study by Flurry et al. described a leech tunnelling into a flap. Across all 17 studies, only five patients did not experience complications.

The association between infection and anaemia and the success of hirudotherapy was subsequently examined. Out of seven total failed flaps, three were infected (OR = 1.8, *p* = 0.646) and four were from patients with anaemia (OR = 5.24, *p* = 0.143).

All eight papers (with a combined sample size of nine) that described infection also reported a causative organism. In six patients (66.67%), Aeromonas Hydrophila was isolated, and in the remaining 3, Aeromonas Veronii was isolated. The average transfusion requirement across both papers (n = 8) reporting anaemia was 5.15 units of pRBC with a range of 1–11 units.

## 4. Discussion

The available literature on the use of leeches in breast surgery is sparse and not directly reported. Despite this, the current systematic review comprising 18 studies and 28 patients represents the first ever systematic review of the evidence of medicinal leeching in breast surgery. It reveals a broad range of insights into the patient cohort, methodology, differential efficacy, and post therapeutic outcomes of hirudotherapy within the context of breast surgery.

Commencing hirudotherapy is a decision that should be made in conjunction with all other appropriate salvage techniques. Firstly, accurate diagnosis of a venous cause of the impending flap failure is paramount. Once this has been established, an understanding of whether the insufficiency relates to obstruction proximal at the venous anastomosis as compared to an internal venous insufficiency within the flap is an important distinction to make. Hirudotherapy is unlikely to be sufficient to salvage an obstruction of proximal venous anastomosis or pedicle obstruction, particularly early before neovascularisation and angiogenesis occur around the flap and surrounding tissues. Often, complete flap venous congestion indicates a more proximal obstruction and is unlikely to be adequately treated by hirudotherapy alone. In free flap surgery, where there are concerns for proximal anastomotic thrombosis, the anastomosis should typically be explored and revised where necessary and leeching could be considered as an adjunctive treatment modality. Moreover, in NAC congestion, the removal of the periareolar skin sutures to exclude haematoma, torsion of the pedicle, and arterial insufficiency should be considered.

Once the decision to begin hirudotherapy is made, the primary concern is determining the appropriate leeching regimen: the number of leeches, frequency, and duration of application. The significant variation in patient cases makes it challenging to establish a standard protocol. Therefore, clinicians should rely on their judgment and case-specific goals to devise these treatment parameters.

In terms of the number of leeches per session, this is dependent on the area and severity of venous insufficiency. Elyassi et al. have attempted to standardise this metric to one leech per 3 cm^2^ of congested skin; however, this is based on head and neck patients and has not been verified in any contexts [29]. Our systematic review found that a median number of 2 leeches (range 1–7) were used per session, which may be appropriate for 6 cm^2^ of congested skin. This is roughly equivalent to half the area of the average nipple areolar complex [30].

The frequency of leech application varies based on the congestion severity, the time it takes for bleeding to stop after leeches detach, and whether the leeches fully engorge with blood before dislodging [31]. The evidence base is varied on this metric with recommendations ranging from hourly to daily [31]. Our systematic review found similarly varied results with a median application rate of three times per day with a range of once daily to once every two hours.

Finally, the decision to discontinue hirudotherapy is based on a multitude of endpoints such as the resolution of congestion and development of complications such as infection, anaemia, or necrosis [31]. To this end, most studies in general plastic surgery claim that hirudotherapy lasts between 2 and 6 days [32,33,34]. Our systematic review found almost identical results with a median of 3 days and range of 1–6 days.

The success of medicinal leeching in breast surgery overall was 75%. It was especially promising in non-reconstructive and pedicle flap contexts when microvascular anastomosis was not conducted. This trend was corroborated by the fact that our review also found that when success was viewed as a subset of aetiology, higher rates of tissue preservation were noted when the venous insufficiency was more distal (venous congestion of flap and venous congestion of NAC). These trends follow physiological reasoning because leeches are especially effective when there are microvenous insufficiencies more distally in a tissue transfer. This is because the draining of the blood from the tissue temporises it until new venous drainage can be formed. This is much less likely to occur when the issue is with a proximal occlusion caused by thrombus formation since no amount of temporisation and drainage will be enough to resolve the thrombus [7]. Furthermore, among our included cases, the salvage of cases of free flaps with venous anastomosis obstruction had typically had anastomotic revisions conducted in theatre prior to the use of leech therapy. Increased exposure to the medicinal leech increases the chance of complications such as infection and anaemia. The literature is largely aligned with this philosophy, where even proponents of hirudotherapy in the salvage of thrombosed flaps concede that there is a large leech burden resulting in frequent and debilitating complications [34].

While these trends hold true when all breast surgeries (both reconstructive and non reconstructive procedures) are examined, a closer inspection of the problematic group of free flaps may reveal some subefficacious practices and beliefs maintained by surgeons using leeches in free flap breast surgery. Ultimately, five out of eight free flaps saw complete flap loss. Amongst these, surprisingly only two failed due to early venous thrombosis—contrary to what one may expect. Therefore, the majority of failed salvages were in instances of venous congestion of the distal skin island of the flap. The counterintuitiveness of this finding could be explained when the individual circumstances surrounding each failed free flap with distal venous congestion are understood. In the deChalain and Pannucci cases, leeches were applied as a last resort measure, where alternative decongestive techniques were attempted, observation for an effect was undertaken, and, failing this, leeches were considered. These practices may be explained by what deChalain describes as a fatalistic impression of leech use in free flap surgery wherein leech use is seen “as an admission of defeat”. Therefore, it follows that this high rate of failure maybe confounded by late and perhaps inappropriately liberal use of leeches in flaps that were bound to fail. This is also seen in the Barraud 2020 case where hirudotherapy was initiated even after part of their flap had begun to necrose, indicating advanced progression of venous insufficiency with the potential for pressure-induced arterial insufficiency. Furthermore, in successful free flap salvages such as those reported by Masters, Flurry, and Baccarini, hirudotherapy was initiated immediately after the identification of congestion or immediately proceeding an attempt at reanastomosis. This lends credence to the notion that leeches used as a useful adjunct to surgical/mechanical decongestion and not as a last resort when all else has failed will result in better outcomes.

It is worth noting that this paper, consistent with the other literature reporting the efficacy of hirduotherapy, defined success as anything outside of complete flap loss. This results in a situation where a patient who only has partial flap salvage after undertaking the physiologically and psychologically demanding ordeal of medicinal leeching may be considered a success where their perception of the outcome may not be so generous.

Complications of hirudotherapy in general plastic surgery are common and well reported. Whittaker et al.’s panoramic systematic review quoted a complication rate of 21.8% across all plastic surgical procedures, which they deemed was already high [3]. Their range of complications was mostly infective, followed by pain, renal injury, and psychosis. The paper also did not include partial skin loss or anaemia requiring transfusion as a complication but reported on it as a separate outcome, differing from the methodology of our systematic review. In our breast surgery population, an 81.14% complication rate was discovered with each patient experiencing 1.45 complications each. The most common of these were infection, partial flap loss, and anaemia.

A third of recorded patients experienced a leech-related infection (28% of all recorded complications). The causative organism was isolated to be exclusively from the Aeromonas genus with a 67% to 33% split between *hydrophila* and *veronii* species. The literature shows an infection rate of 7–20% in all plastic surgery cases with the predominant organism also being from the Aeromonas genus [35]. Noting that the reviewed population did not on average have a more frequent or longer period of hirudotherapy, this may represent a non-confounded increase in the rate of infection within the analysed breast population. Furthermore, the failure of hirudotherapy was overrepresented in patients who developed infection, though it is difficult to ascertain whether this was infection driving failure or the necessity for a more rigorous leeching protocol in failing flaps driving infection.

It is common practice to provide patients with prophylaxis against Aeromonas species with a fluroquinolone such as ciprofloxacin. However, this review [17,21,26] suggests that this is becoming inappropriate cover due to the increasing prevalence of multidrug-resistant Aeromonas species. Our population most frequently required targeted antibiotic therapy with aminoglycosides, second-generation cephalosporins, and metronidazole.

Anaemia requiring transfusion was another common complication. On average, our population required 5.15 units of pRBCs with a range of 1–11 units. This is not uncommon in the literature; a series of eight congested head and neck flaps treated with leeches required 13 units of pRBCs on average to resolve anaemia [34]. In all cases, anaemia was associated with increased leech burden. However, postoperative anaemia in the context of breast surgery is likely to be multi-factorial including intraoperative blood loss or postoperative haematoma. Furthermore, this review saw an overrepresentation of failure of tissue transfer within patients who developed anaemia. Once again, it is difficult to determine the order of this association, but it may be more logical to assume that failing flaps had more significant venous congestion necessitating more intense hirudotherapy leading to anaemia. Each transfusion carries with it risks such as cross reactivity and infection. Additionally, periods of anaemia place added cardiorespiratory stress on a patient who has already undergone major surgery [36]. Moreover, bleeding around the leech site may promote a favourable environment for infection, increasing the risk of soft tissue infection as previously mentioned. As such, the judicious clinician must be cognisant of the extent to which they are willing to proceed to support their hirudotherapy.

Practically, this systematic review underscores the necessity for clinician discretion when deciding to engage in medicinal leeching in breast surgery. It highlights that the quantity, frequency, and time course of hirudotherapy should be closely dependent on the size of cutaneous venous congestion, its severity, and the presence of complications and favourable endpoints. It also demonstrates that medicinal leeching is much more effective in resolving venous congestion that is distally based such as in NAC congestion and non-thrombotic free flap venous insufficiency. As such, hirudotherapy may be more encouraging in these cases than in instances of free flap venous thrombosis. Finally, the decision to undertake hirudotherapy is closely associated with the need to balance the burden of complications. Infection should be closely watched and appropriately protected against with the aid of a microbiology team and anaemia should be avoided where possible. Novel, chemical, and mechanical alternatives to leeching may also be considered in patient cases where infection is unacceptable [37].

The weaknesses of this review are based mostly on the dearth of evidence that exists on this topic. This results in a review with a relatively low sample size and no descriptors of variance in the data. As such, meta-analyses could not be undertaken, meaning that inferences regarding these data are only technically valid in the context of the discussed studies. Therefore, there may be variability in accuracy when the recommendations garnered from this study are applied to a new clinical context. Furthermore, the mechanism of associations that are discovered in this review, such as those between the aetiology of venous insufficiency and the success of hirudotherapy, can only be hypothesised and remain uncertain. Finally, to increase sample size as much as possible, studies that examine both reconstructive and non-reconstructive breast surgery were included. This introduces additional heterogeneity in the interpretations made from these data. As such, the goal of this paper is to further encourage more experimentally sound, larger scale undertakings in hirudotherapy in breast surgery. An increased number of case series, case control studies, and potentially randomised controlled trials may permit the amelioration of the current paper’s drawbacks.

## 5. Conclusions

This systematic review comprising 18 papers and 28 patients demonstrated an overall success rate of 75% when medicinal leeches were used within breast surgery to resolve venous congestion. While this suggests that medicinal leeching shows promise in resolving venous insufficiency, this optimism must be tempered with consideration for overall patient satisfaction. This review demonstrated that hirudotherapy may be more effectively used when initiated early and in instances of distally based venous congestion and that it carried a complication rate as high as 81.14%. Consequently, careful clinical judgment is crucial for the effective and successful application of hirudotherapy in breast surgery.

## Figures and Tables

**Table 1:** Characteristics of Included Studies.

Year	Author	Country	Study Type	n	Age	Procedures	Indication for Hirudotherapy	Prior Salvage	LPS	LPD	LOH(D)	Success (n)	(%)
1984	Dickson et al. [11]	UK	Case Report	1	27	Pedicled flap	Venous Congestion of Flap	Nil	NR	NR	4	1	100%
1992	Gross et al. [12]	USA	Case Series	2	Case 1: 20	Mastopexy	Venous Congestion of NAC	Nil	1	3	3	2	100%
					Case 2:26	Reduction Mammoplasty	Venous Congestion of NAC	Nil	2	1	2		
1992	Lineaweaver [13]	USA	Case Report	1	34	Pedicled Flap	Venous Congestion of Flap	Nil	3	NR	3	1	100%
1996	De Chalain et al. [14]	USA	Case Series	6	Case 1: 28	Free Flap	Venous Thrombosis of Anastomosis	Theatre	NR	NR	1.5	4	66.67%
					Case 2: 44	Pedicled Flap	Venous Congestion of Flap	Nil	NR	NR	3		
					Case 3: 35	Pedicled Flap	Venous Congestion of Flap	Nil	NR	NR	2		
					Case 4: 52	Free Flap	Venous Thrombosis of Anastomosis	Nil	NR	NR	1		
					Case 5: 56	Free Flap	Venous Thrombosis of Anastomosis	Theatre	NR	NR	4		
					Case 6: 41	Free Flap	Venous Congestion of Flap	Theatre	NR	NR	3		
2000	Guneren et al. [15]	Turkey	Case Report	1	24	Reduction Mammoplasty	Venous Congestion of NAC	Nil	4	3	3	1	100%
2002	Spera [16]	USA	Case Report	1	39	Reduction Mammoplasty	Venous Congestion of NAC	Nil	4	2	2	1	100%
2004	Ardehali et al. [17]	UK	Case Study	1	47	Pedicled Flap	Venous Congestion of Flap	Nil	NR	NR	4	1	100%
2009	Camara et al. [18]	Germany	Case Study	1	NA	Free Flap	Venous Congestion of Flap	NR	3	NR	NR	1	100%
2009	Bourdais et al. [19]	France	Case Study	1	56	Pedicled Flap	Venous Congestion of Flap	Nil	NR	NR	5	0	0%
2011	Flurry et al. [20]	USA	Case Report	1	40	Free Flap	Venous Congestion of Flap	Nil	1	NA	2	1	100%
2012	Maetz et al. [21]	France	Case Series	2	Case 1: 55	Pedicled Flap	Venous Congestion of Flap	Theatre	NR	NR	4	2	100%
					Case 2: 56	Pedicled Flap	Venous Congestion of Flap	Nil	NR	NR	3		
2014	Pannucci et al. [22]	USA	Case Series	4	Case 1: 45.8	Free Flap	Venous Congestion of Flap	Theatre	1	6	3	1	25%
					Case 2: 64.4	Free Flap	Venous Thrombosis of Anastomosis	Theatre	7	12	6		
					Case 3: 58.3	Free Flap	Venous Congestion of Flap	Nil	2	12	3		
					Case 4: 28.3	Free Flap	Venous Thrombosis of Anastomosis	Nil	2.75	4	4		
2015	Freeman et al. [23]	USA	Case Study	1	61	Reduction Mammoplasty	Venous Congestion of NAC	Nil	2	3	3	1	100%
2015	Moffat et al. [24]	USA	Case Report	1	34	Reduction Mammoplasty	Venous Congestion of NAC	Pharm	2	3	3	1	100%
2019	Barraud et al. [25]	France	Case Study	1	NA	Free Flap	Venous Congestion of Flap	Nil	NS	NS	1	0	0%
2020	Masters et al. [26]	USA	Case Report	1	50	Free Flap	Venous Congestion of Flap	Nil	2	NR	3	1	100%
2021	Vasei et al. [27]	Iran	Case Report	1	23	Reduction Mammoplasty	Venous Congestion of NAC	Pharm	5	1	1	1	100%
2022	Baccarani et al. [28]	Italy	Case Study	1	42	Free Flap	Venous Congestion of Flap	Theatre	2	NR	3	1	100%

USA = United States of America, UK = United Kingdom, NAC = nipple areolar complex, LPS = number of leeches per session, LPD = leechings per day, LOH = length of hirudotherapy (days), NR = not recorded, NA = not applicable. NS= not significant.

**Table 2:** Details of complications among included studies.

Year	Author	n	Complications	Infectious Organism	Pharmacological Adjuvants	Transfusion (Units of PRBC)
1984	Dickson et al. [11]	1	Partial Flap Loss; Infection	A. Hydrophila	Unspecified Antibiotics	Nil
1992	Gross et al. [12]	2	Nil	Nil	Nil	Nil
			Partial Flap Loss	Nil	Nil	Nil
1992	Lineaweaver [13]	1	Partial Flap Loss; Infection	A. Hydrophila	Penicillin; Cefoxitin	Nil
1996	De Chalain et al. [14]	6	Partial Flap Loss; Anaemia	Nil	Nil	11
			Total Flap Loss; Anaemia; Infection	A. Hydrophila	Clindamicin; Ciprofloxacin	3
			Partial Flap Loss	Nil	Nil	Nil
			Total Flap Loss	Nil	Nil	Nil
			Anaemia	Nil	Nil	6
			Anaemia	Nil	Nil	1
2000	Guneren et al. [15]	1	Nil	Nil	HMW Dextran	Nil
2002	Spera [16]	1	Nil	Nil	Nil	Nil
2004	Ardehali et al. [17]	1	Partial Flap Loss; Infection	A. Hydrophila	Amoxycilin Clauvanic Acid	Nil
2009	Camara et al. [18]	1	Nil	Nil	Nil	Nil
2009	Bourdais et al. [19]	1	Total Flap Loss; Infection	A. Hydrophila	Ciprofloxacin; Gentamicin; Metronidazole	Nil
2011	Flurry et al. [20]	1	Leeches Tunnelling into Flap	Nil	Nil	Nil
2012	Maetz et al. [21]	2	Infection	A. Veronii	Vancomycin; Cefotaxime; Clindamicin	Nil
			Partial Flap Loss; Infection	A. Veronii	Amoxycilin Clauvanic Acid; Gentamicin	Nil
2014	Pannucci et al. [22]	4	Parial Flap Loss; Anaemia	Nil	Nil	6
			Total Flap Loss; Anaemia	Nil	Nil	10
			Total Flap Loss; Anaemia	Nil	Nil	2
			Total Flap Loss; Anaemia	Nil	Nil	2
2015	Freeman et al. [23]	1	NR	NR	NR	NR
2015	Moffat et al. [24]	1	Nil	Nil	HBO2; GTN; Pentoxyfylline	Nil
2019	Barraud et al. [25]	1	Total Flap Loss; Infection	A. Veronii	Cefepime; Metronidazle; Gentamicin	Nil
2020	Masters et al. [26]	1	Infection	A. Hydrophila	Carbapenem	NR
2021	Vasei et al. [27]	1	Partial Flap Loss	Nil	Nil	Nil
2022	Baccarani et al. [28]	1	Nil	Nil	Heparin; Dexamethasone	Nil

NR = not recorded, PRBCs = packed red blood cells.

**Table 3:** Quality assessment of included studies using the Joanna Briggs Institute (JBI) Critical Appraisal Checklist for Case Series.

Year	Author	Q1	Q2	Q3	Q4	Q5	Q6	Q7	Q8	Q9	Q10
1984	Dickson et al. [11]	Y	Y	Y	N	N	Y	U	Y	Y	NA
1992	Gross et al. [12]	Y	Y	Y	Y	Y	Y	U	Y	Y	NA
1992	Lineaweaver [13]	Y	Y	Y	N	N	Y	U	Y	Y	NA
1996	De Chalain et al. [14]	Y	Y	Y	Y	Y	Y	Y	Y	Y	Y
2000	Guneren et al. [15]	Y	Y	Y	N	N	Y	Y	Y	Y	NA
2002	Spera [16]	Y	Y	Y	N	Y	Y	U	Y	Y	NA
2004	Ardehali et al. [17]	Y	Y	Y	N	N	Y	N	Y	Y	NA
2009	Camara et al. [18]	N	N	Y	Y	Y	Y	Y	Y	Y	Y
2009	Bourdais et al. [19]	Y	Y	Y	Y	N	N	Y	Y	Y	Y
2011	Flurry et al. [20]	Y	Y	Y	N	N	Y	U	Y	Y	NA
2012	Maetz et al. [21]	Y	Y	Y	Y	Y	Y	U	Y	Y	NA
2014	Pannucci et al. [22]	Y	Y	Y	Y	N	Y	Y	Y	Y	Y
2015	Freeman et al. [23]	Y	Y	Y	N	N	Y	Y	Y	Y	NA
2015	Moffat et al. [24]	Y	U	Y	N	N	Y	N	Y	Y	NA
2019	Barraud et al. [25]	Y	Y	Y	Y	Y	Y	Y	Y	Y	Y
2020	Masters et al. [26]	Y	Y	Y	N	N	N	Y	Y	Y	NA
2021	Vasei et al. [27]	Y	Y	Y	N	N	Y	Y	Y	Y	NA
2022	Baccarani et al. [28]	Y	Y	Y	N	N	Y	Y	Y	Y	NA

Y = Yes, N = No, U = Unclear, NA = Not Applicable.

## Data Availability

Not applicable.
